# Efficacy of 3D‐Printed Cleaning Splints for Approximal Dental Biofilm Removal—A Randomized Clinical Crossover Pilot Study

**DOI:** 10.1002/cre2.70392

**Published:** 2026-06-23

**Authors:** Marcia Spindler, Theresa Becker, Stefan Rues, Kevin Richter, Peter Rammelsberg, Andreas Zenthöfer, Diana Wolff, Valentin Bartha

**Affiliations:** ^1^ Department of Conservative Dentistry, Heidelberg Faculty of Medicine Heidelberg University Heidelberg Germany; ^2^ Department of Prosthetic Dentistry, Heidelberg Faculty of Medicine Heidelberg University Heidelberg Germany

**Keywords:** 3D‐printed splints, biofilm removal, interdental brushes, periodontitis

## Abstract

**Objectives:**

This study investigated the effectiveness of biofilm removal using individually customized cleaning splints (CS) as an aid for interdental brushes (IDBs).

**Materials and Methods:**

In a randomized clinical crossover study, the effect of IDBs in combination with CS on plaque reduction and periodontal parameters was examined. Periodontitis patients received an intraoral scan to produce a 3D‐printed CS. Following a 2‐week interdental hygiene pause, they were divided into two groups: Group A: CS+IDBs, Group B: IDBs. Participants performed daily interdental cleaning at home for 2 weeks following standardized professional instruction. After another 2‐week interdental hygiene pause, the groups switched methods. Additionally, motor skills, stress levels, and nutritional quality were assessed.

**Results:**

Thirty participants (age range 21–82 years) completed the study. CS+IDBs led to a significantly greater plaque reduction (change in Quigley–Hein plaque index, ∆QHI) compared to IDBs alone (ΔQHI = −1.38 vs. −0.23, *p* < 0.001). The impact on reducing gingival inflammation (GI) was limited to younger participants with no effect on bleeding on probing (BOP). Random effects ANCOVA confirmed the significant effect of CS on ∆QHI (*p* < 0.001). An interaction between perceived stress and CS use on BOP indicated that stress may influence the effectiveness of CS supported plaque removal.

**Conclusions:**

Cleaning splints are a promising approach to enhance the effectiveness of IDBs in reducing interdental biofilm.

## Summary

**Background:**

Interdental brushes (IDBs) remain the gold standard for mechanical interdental plaque control. However, many patients face difficulties with their use and handling.

**Added Value:**

In this randomized clinical crossover trial, customized 3‐D printed cleaning splints (CS) were tested as an adjunct to IDBs in periodontitis patients. The combination of CS and IDBs significantly improved interdental plaque removal compared with IDBs alone, highlighting their potential to facilitate patient handling and enhance daily biofilm control.

**Clinical implications:**

Cleaning splints offer a patient‐specific adjunct for optimizing interdental hygiene in periodontitis patients and could also be a valuable tool for patients in nursing homes or other vulnerable groups.

## 1 Introduction

Periodontal disease and dental caries are among the most prevalent and most costly oral health conditions (Eickholz et al. 2025; Jevdjevic and Listl 2025; Jordan et al. 2025). In Germany, the prevalence of periodontitis is high among younger adults and younger seniors, with severe forms (stages III and IV) affecting 17.5% and 52.7% of these age groups, respectively (Eickholz et al. 2025). Both periodontal disease and caries are associated with dysbiotic changes within dental biofilms, influenced by ecological and host factors (Marsh 2006; Socransky and Haffajee 2005).

Despite evidence that several lifestyle factors, such as dietary changes, can influence the severity of dental diseases, the mechanical disruption of oral biofilm remains an essential part of prevention and treatment strategies (Worthington et al. 2019). Toothbrushing alone cannot effectively reach the interdental areas, where plaque accumulates more rapidly and is more prevalent, contributing to a higher incidence of periodontal diseases in these sites (Christou et al. 1998; Cumming and Löe 1973; Löe et al. 1965).

For interdental hygiene, interdental brushes (IDBs) are preferred due to their superior efficacy compared to other aids, with the greatest effect in reducing gingivitis (Sälzer et al. 2015; Sanz et al. 2020). However, interdental cleaning and the manual handling of IDBs present challenges for many patients (Slot et al. 2008). In particular, finding the correct position for IDB insertion, brush bending, distortion and clotting, where overlapping filaments during insertion increase the brush's incompressible diameter, raise the risk of incorrect insertion and potential trauma to the gingival soft tissues (Wolff et al. 2006). Especially in cases where guide surfaces are absent, such as in partially edentulous jaws, dentures, and missing adjacent teeth, as well as in large interdental spaces, adequate cleaning of approximal tooth or implant surfaces is particularly difficult, and IDBs may not reliably reach the critical approximal cervical areas without guidance (Kern 2012; Passia and Kern 2021).

In these situations, a cleaning splint (CS) can enhance the effectiveness of approximal oral hygiene measures (Kern 2012; Passia and Kern 2021). Cleaning splints can facilitate the insertion and guidance of IDBs, especially in cervical areas. This approach may be particularly beneficial for patients with dentures, implants, teeth with missing guide surfaces, and those with manual limitations (Kern 2012). However, there is currently no clinical data available on their cleaning efficacy.

Therefore, a recent in vitro study revisited and further developed this concept to assess cleaning efficacy with and without CS, demonstrating a significantly improved approximal cleaning efficacy for a partially edentulous typodont model compared to cleaning without the CS (Rues et al. 2024).

These effects of CS on interdental cleaning in an experimental model support the assumption that creating guide openings for interdental brushes can enhance mechanical biofilm removal from the approximal surfaces of teeth and retention elements of prosthetic restorations in patients. Specifically, for terminal teeth or cases with missing adjacent teeth in partially edentulous jaws, defined guide surfaces may improve cleaning effectiveness. However, clinical studies validating the efficacy of CS in practice are still lacking. Therefore, this study aimed to assess the impact of customized cleaning splints on the efficacy of IDBs for biofilm removal in a clinical setting. The reduction of plaque was considered the primary outcome, while effects on periodontal bleeding parameters were examined as secondary outcomes. Therefore, the null hypothesis was that the use of a customized cleaning splint in combination with interdental brushes would not result in greater plaque reduction or improvements in periodontal parameters compared to interdental brushes alone.

## 2 Materials and Methods

This randomized controlled clinical study used a parallel crossover design to assess the effectiveness of customized CS in enhancing interdental biofilm removal. Ethical approval was obtained from the Ethics Committee of the Medical Faculty at Heidelberg University prior to participant recruitment (Approval No. S‐387/2023). The study was conducted and reported in accordance with the Consolidated Standards of Reporting Trials (CONSORT) 2025 guidelines and registered in the German Clinical Trials Register (DRKS, ID: DRKS00034674, https://drks.de/search/de/trial/DRKS00034674/details). No patients or patient representatives were involved in the design, conduct, or reporting of this trial.

### 2.1 Study Population

Adults diagnosed with periodontitis were recruited at the Department of Conservative Dentistry, Heidelberg University, Heidelberg, Germany, between October 2023 and August 2024. Inclusion criteria required informed consent and the presence of at least two teeth within one jaw. Participants were grouped by age: ≤ 40 and ≥ 65 years.

Exclusion criteria included antibiotic use within the past 3 months and medications and severe systemic conditions, for example, high endocarditis risk, immunosuppression, or infectious diseases like HIV.

Collected demographic and medical data included age, gender, and smoking status (current, former, non‐smoker). Manual dexterity was tested using the Goldenberg Apraxia Test (Goldenberg 2006). Dietary habits and stress levels were evaluated using the Mediterranean Diet Adherence Screener (MEDAS) (Hebestreit et al. 2017) and the Perceived Stress Scale (PSS‐10) (Cohen et al. 1983).

No important changes to the trial protocol, prespecified outcomes, or planned analyses were made after the study commenced. One participant did not complete the intervention, and therefore an additional participant was recruited to maintain the planned sample size.

### 2.2 Study Design

**Figure 1:**
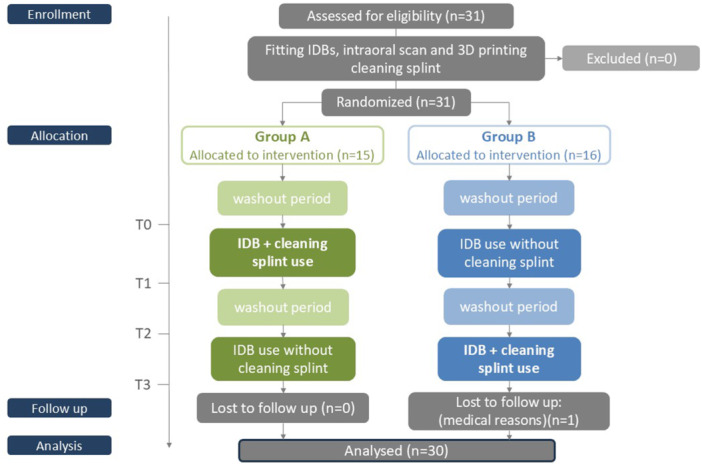
Flowchart of the crossover study design. Participants were randomly allocated to two groups (A and B), each serving as both the experimental and control group at different time points. The study followed CONSORT guidelines and included baseline assessments, a 2‐week washout period, and 2‐week sequential intervention phases, with clinical evaluations conducted at multiple time points (T0–T3).

Participants were randomly assigned to two groups (A and B), each serving as both the experimental and control group at different time points (Figure 1). Blinding was not feasible; therefore, unblinded block randomization stratified by age was performed using sealed envelopes.

### 2.3 Baseline Examination and Preparation

Standardized oral hygiene instructions were given, and participants practiced using the splint. Cylindrical interdental brushes (Curaprox, Curaden AG, Kriens, Switzerland) were individually selected based on the clinically assessed passage hole diameter (PHD) of each interdental space. Multiple brush sizes were assigned per participant according to regional interdental morphology, with a maximum of three different interdental brush sizes provided per individual. Participants were instructed to insert the interdental brush using a straight in‐and‐out motion without additional adaptation along the tooth surface. The correct handling technique was demonstrated and individually practiced until a sufficient level of competence was achieved under supervision. Participants completed the German version of the MEDAS and of the Perceived Stress Score (PSS‐10) to evaluate possible differences in systemic modulatory effects of periodontal inflammation. Additionally, the Goldenberg Apraxia Test was completed by each participant, aiming to evaluate differences in motoric skills. Intraoral scans were taken (Primescan, Dentsply Sirona, Bensheim, Germany) and 3D‐printed cleaning splints were fabricated (Freeprint Splint 2.0, Detax, Ettlingen, Germany).

During a 2‐week washout period, oral hygiene was standardized: participants used Elmex toothpaste (Elmex, CP GABA, Switzerland) and refrained from additional oral hygiene aids (e.g., interdental brushes, antibacterial rinses). Participants were instructed to maintain their usual toothbrushing habits throughout the study period.

### 2.4 Intervention and Clinical Assessments

Upon completion of the CS, participants were randomly allocated in group A or B. All clinical examination parameters were assessed by one single examiner. At Baseline (T0, after the first washout period), the assessed periodontal parameters included probing pocket depth (PPD), clinical attachment level (CAL), bleeding on probing (BOP), and oral hygiene indices (Quigley‐Hein plaque index [QHI] [Turesky et al. 1970], Gingival Index [GI] [Löe and Silness 1963]) at 4 sites per tooth mesial and distal.

**Group A: Cleaning Splint First**

- Participants received customized CS, which were checked for fit and comfort, and IDB sizes were re‐evaluated for compatibility.
- Standardized oral hygiene instructions were given, and participants practiced using the splint.
- Participants used the splints for 2 weeks, followed by a reassessment of clinical parameters (T1).
- A 2‐week washout phase followed, prohibiting interdental hygiene. At the end of this second washout phase (T2), clinical parameters were remeasured.
- Participants resumed using IDBs without CS for 2 weeks. At the end (T3), final assessments took place.

**Group B: Cleaning Splint Second**

In group B, CSs were used between T2 and T3, while between T0 and T1 the participants cleaned without CSs. All examinations and washout periods were equal between the groups.

The primary endpoint was the QHI as a clinical measure of plaque accumulation. Secondary endpoints included PPD, BOP, CAL, and GI. No adverse events or harms were anticipated or observed during the study.

### 2.5 Cleaning Splint Manufacturing

**Figure 2:**
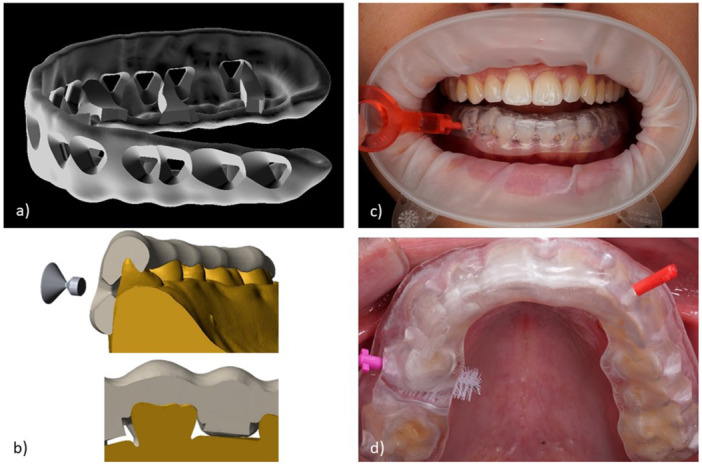
(a) Computer‐aided design (CAD) of the customized cleaning splint, featuring funnel‐shaped openings with triangular cross‐sections. The design includes guide surfaces strategically placed adjacent to terminal teeth and edentulous spaces to facilitate interdental cleaning; (b) cross‐section of the splint; (c) intraorally positioned cleaning splint in a frontal view, with the patient inserting an interdental brush into a guide opening, photo: Dr. Sven Räther, 2024; (d) occlusal view of the cleaning splint. It can be observed that the interdental brushes reach the oral side along their full length, with the splint not obstructing their insertion.

Based on digital scans, a customized CS was fabricated using 3D printing technology. Small, funnel‐shaped guide openings were incorporated at interdental spaces to facilitate the insertion of IDB. IDB insertion axes for all interdental spaces were given by the highest points of the papillae on the vestibular and oral side. At the narrowest part of each opening with a triangular cross‐section, the radius of the triangle's incircle was 0.5 mm larger than the respective passage hole diameter. Additionally, guide surfaces were integrated along terminal teeth or surfaces lacking adjacent teeth to ensure a consistent trajectory of the interdental brush along the approximal curvature (Figure 2). In terminal teeth, smaller IDBs were selected compared to teeth adjacent to edentulous spaces. The distance between the guide area and the tooth surface was set at either 1.0 mm or 2.0 mm. Furthermore, to facilitate IDB insertion for the distal surface of terminal teeth, the respective opening was manually rotated in the mesial direction. The manufacturing procedure has been described in detail in a prior in‐vitro investigation (Rues et al. 2024).

### 2.6 Statistical Analysis

This study was conducted as a pilot study with an exploratory analysis, with sample size determination based primarily on feasibility considerations, including recruitment capacity and logistical aspects rather than a formal a priori power estimation. Although preliminary in vitro data were available, these findings were considered insufficient for a reliable estimation of clinical effect sizes due to the limited transferability of in vitro results to the clinical setting and the expected interindividual variability among participants. In this context, all *p*‐values should be interpreted descriptively. No interim analyses or stopping guidelines were planned or implemented. No missing data occurred; therefore, no imputation methods were required. Given the focus on interdental cleaning efficacy and to be able to identify effects of missing adjacent teeth and tooth regions on cleaning efficacy, the approximal space was defined as the primary statistical unit. To account for the hierarchical structure of the data, a two‐step aggregation approach was applied: First, individual measurements per interdental space were averaged to obtain a single value per approximal space. Second, the approximal space‐level means were aggregated at the patient level for inter‐individual patient‐level comparisons. Potential carry‐over effects that arise from the interaction of treatment and time were evaluated by comparing the summed values during test‐ and control‐intervention between groups A and B. At the patient and approximal space level, no effects were observed. Based on these results, further investigations were carried out for the pooled data of both groups.

Statistical comparisons between the test intervention (cleaning splint) and the control intervention (interdental cleaning without the splint) were performed using Wilcoxon signed‐rank tests for intra‐group comparisons and Mann–Whitney *U* tests for inter‐group comparisons.

To adjust for potential confounders and account for repeated measurements within individuals, an ANCOVA with a random effects model was applied. Patient ID was included as a random effect to model intra‐individual dependencies and control for variation across subjects. Fixed effects included intervention type (test vs. control), baseline values, apraxia score, MEDAS, and PSS. Additionally, interaction terms were introduced to assess modifying effects, particularly regarding tooth gaps and anterior versus posterior tooth regions. All planned analyses, including subgroup analyses were pre‐specified in the study protocol.

All statistical tests were performed using SAS JMP, version 18.2.1 (SAS Institute, Heidelberg, Germany) with a significance level of *p* < 0.05.

## 3 Results

Out of 31 participants, a total of 30 participants (11 females) with a mean age of 52.5 ± 21.7 years (median 53 years, interquartile range 31.8–73; range 21–82) completed the study. One participant dropped out due to a cardiovascular event (Figure 1). Age group 1 (AG1) (≤ 40 years) included 6 females and 9 males (mean age 31.7 ± 6.1 years), and age group 2 (AG2) (≥ 65 years) included 5 female and 10 male participants (mean age 73.3 ± 4.1 years).

In total, six participants were smokers. The MEDAS yielded an average score of 5.6 ± 1.6 out of a possible 14 points, with no significant differences between the age groups. The mean PSS score was 14.0 ± 4.5, with a trend toward higher values in AG1; however, this difference was marginally non‐significant (*p* = 0.055).

A significantly lower apraxia score was observed in AG2 (53.6 ± 1.9) compared to AG1 (55.0 ± 0.0, *p* = 0.001), where all participants achieved the maximum value (Table 1).

**Table 1:** Baseline characteristics of all patients and age group 1 (≤ 40 years) as well as age group 2 (≥ 65 years). *p*‐values by Mann–Whitney *U* test for continuous variables and by Chi‐square test for categorical variables.

	All		Age group 1		Age group 2		*p* value
Female (*n*, %)	11	37%	6	40%	5	33%	0.704
Male (*n*, %)	19	63%	9	60%	10	67%	
Smoker (*n*, %)	6	20%	4	29%	2	13%	0.311
Age (mean, SD)	52.53	±21.76	31.73	±6,05	73.33	±4.12	**< 0.001**
MEDAS (mean, SD)	5.57	±1.57	5.53	±1.81	5.60	±1.35	0.932
PSS (mean, SD)	14.01	±4.46	15.41	±3.95	12.61	±4.64	0.055
Apraxia score (mean, SD)	54.30	±1.51	55.00	±0.00	53.6	±1.91	**0.001**
Number of teeth (mean, SD)	23.80	±5.30	27.20	±2.10	20.40	±5.80	**< 0.001**
Interdental surfaces (mean, SD)	42.70	±11.30	50.80	3.10	34.60	±12.10	**< 0.001**
Edentulous spaces (mean, SD)	1.13	±1.61	0.27	0.59	2.00	±1.87	**0.002**
Periodontal Stage							**< 0.001**
Stage I	5	16.7	5	33.3	0	0	
Stage II	7	23.3	6	40.0	1	6.7	
Stage III	11	36.7	4	26.7	7	46.7	
Stage IV	7	23.3	0	0	7	46.7	
Periodontal Grade							**0.002**
Grade A	7	23.3	7	46.7	0	0	
Grade B	21	70.0	6	40.0	15	100	
Grade C	2	6.7	2	13.3	0	0	
Extend							**< 0.001**
Localized	14	46.7	12	80.0	2	13.3	
Generalized	16	53.3	3	20.0	13	86.7	
Treatment status							**0.001**
APT	4	13.3	4	26.7	0	0.0	
SPC	26	86.7	11	73.3	15	100	
Periodontal baseline parameters							
PD (mm)	3.01	0.64	3.05	0.83	2.96	0.39	0.77
CAL (mm)	3.58	1.07	3.13	1.26	4.03	0.58	**< 0.001**
PD ≥ 5 mm (n)	20.57	19.25	21.8	24.52	19.33	12.75	0.59
PD ≥ 5 mm (%)	21.37	17.33	19.73	21.18	23	12.95	0.15

*Note:* Significant *p*‐values are presented in bold.

Abbreviations: APT, active periodontal therapy (steps 1 and 2); CAL, clinical attachment loss; MEDAS, Mediterranean Diet Adherence Screener; PD, probing depth; PSS, perceived stress score; SPC, supportive periodontal care (step 4).

The dentition status varied considerably between participants. The mean number of teeth was 23.8 ± 5.3, with significantly higher values in AG1 compared to AG2 (*p* < 0.001). Accordingly, the number of interdental surfaces was significantly greater in AG1 (*p* < 0.001), while edentulous spaces were more frequent in AG2 (*p* = 0.002).

Regarding periodontal classification, significant differences between age groups were observed. Higher stages of periodontitis were predominantly found in AG2 (*p* < 0.001). Similarly, grade distribution differed significantly, with AG1 showing more Grade A cases, whereas AG2 was dominated by Grade B (*p* = 0.002). The extent of periodontitis also differed markedly, with localized disease being more frequent in AG1 and generalized forms in AG2 (*p* < 0.001).

Most participants had already undergone active periodontal therapy (86.7%). While all participants in AG2 were treated, a proportion of AG1 participants were still untreated; however, this difference did not reach statistical significance when assessed with Fisher's exact test.

Baseline periodontal parameters revealed a significantly higher clinical attachment loss (CAL) in AG2 compared to AG1 (*p* < 0.001). In contrast, mean probing depths and the number as well as proportion of sites with increased probing depth did not differ significantly between groups.

No harms or unintended events related to the interventions occurred in either group.

### 3.1 Analysis of Potential Carry‐Over‐Effects Between the Two Parallel Groups

Combined values for QHI, GI, and BOP (test and control interventions) revealed no significant differences between Groups A and B (Mann–Whitney *U* test), indicating no carry‐over effects. For the primary outcome parameter QHI, median change scores did not differ between groups (∆QHI: Group A −0.73 [Q1: −1.34; Q2: −0.19]; Group B −0.5 [Q1: −1.49; Q2: −0.16]; *p* = 0.964).

The effect of the CS was then assessed by comparing test (CS) (Group A: T0, T1; Group B: T2, T3) and control intervention (interdental cleaning without CS) (Group A: T2, T3; Group B: T0, T1) across both groups.

### 3.2 Changes in QHI With and Without the Cleaning Splint

Overall, the QHI decreased significantly during each cleaning period, regardless of whether the CS was used (test intervention: median ∆QHI = −1.38, *p* < 0.001) or not (control intervention: median ∆QHI = − 0.23, *p* = 0.002).

In contrast, within AG1, a statistically significant QHI reduction was found only when CS was used (median −1.23, *p* < 0.001). In AG2, both test and control treatments led to significant QHI reductions, similar to the overall cohort.

A comparison of the 2‐week (2W) values between the test and control interventions showed statistically significantly lower QHI values for the test intervention in both the overall cohort and the two age groups (Table 2).

**Table 2:** Clinical parameters at baseline and after 2 weeks with (CS+) and without (CS−) cleaning splint. Values are median (Q1–Q3). *p*‐values from the Wilcoxon signed‐rank test (within) and the Mann–Whitney *U* test (between).

Group	Parameter	CS+ baseline (median [Q1–Q3])	CS + 2W (median [Q1–Q3])	p (CS+)	CS− baseline (median [Q1–Q3])	CS − 2W (median [Q1–Q3])	p (CS−)	p (2W intergroup)
All	QHI	3.15 (2.65–3.62)	1.48 (1.26–1.79)	< 0.001	3.07 (2.72–3.46)	2.87 (2.35–3.15)	0.002	**< 0.001**
	GI	0.26 (0.13–0.37)	0.13 (0.06–0.27)	< 0.001	0.31 (0.20–0.71)	0.24 (0.17–0.44)	0.089	**0.002**
	BOP (%)	12.77 (5.60–17.06)	6.27 (2.44–8.71)	< 0.001	11.25 (5.08–23.38)	9.83 (4.72–17.27)	0.078	0.034
AG1 (≤ 40)	QHI	2.80 (1.92–3.23)	1.46 (0.83–1.92)	< 0.001	2.97 (1.88–3.18)	2.74 (1.68–3.06)	0.168	**0.006**
	GI	0.27 (0.13–0.33)	0.13 (0.07–0.27)	0.010	0.27 (0.20–0.37)	0.25 (0.20–0.43)	0.434	0.011
	BOP (%)	16.67 (8.33–17.50)	6.67 (5.00–8.33)	0.008	19.17 (10.83–23.53)	12.50 (8.33–19.85)	0.251	0.020
AG2 (≥ 65)	QHI	3.42 (3.05–4.09)	1.59 (1.32–1.73)	< 0.001	3.37 (2.98–3.69)	2.89 (2.72–3.21)	0.005	**< 0.001**
	GI	0.25 (0.13–0.58)	0.13 (0.00–0.29)	0.002	0.44 (0.19–0.75)	0.19 (0.08–0.48)	0.003	0.080
	BOP (%)	10.71 (5.36–14.00)	4.35 (1.67–8.87)	0.002	6.25 (4.17–16.35)	5.21 (3.33–13.04)	0.193	0.213

*Note:* Significant *p*‐values are presented in bold.

Abbreviations: 2W, 2 weeks time point; AG1, age group 1 (18–40 years); AG2, age group 2 (≥ 65 years); BOP, bleeding on probing; CS+, cleaning splint was used; CS−, cleaning splint was not used; GI, Gingiva Index; med, median; QHI, Quigley–Hein Index; SD, standard deviation.

### 3.3 Changes in GI and BOP

Use of the CS led to a significant reduction in GI (median −0.13) and BOP (median −4.3%) during the cleaning period (*p* < 0.001). In contrast, no significant change in GI or BOP was observed for the control intervention.

CS use significantly reduced GI (*p* = 0.002) and BOP (*p* = 0.034) in the overall cohort. However, this effect was only observed for the reduction in GI within AG1 and did not reach statistical significance for any inflammatory parameter within AG2.

Similar to QHI, comparing the 2 W values of the test and the control intervention, a significantly lower GI and BOP was found in AG1. This effect was not observed in AG2 (Table 2).

### 3.4 Effects of the Cleaning Splint on the Reduction of QHI, GI, and BOP

**Figure 3:**
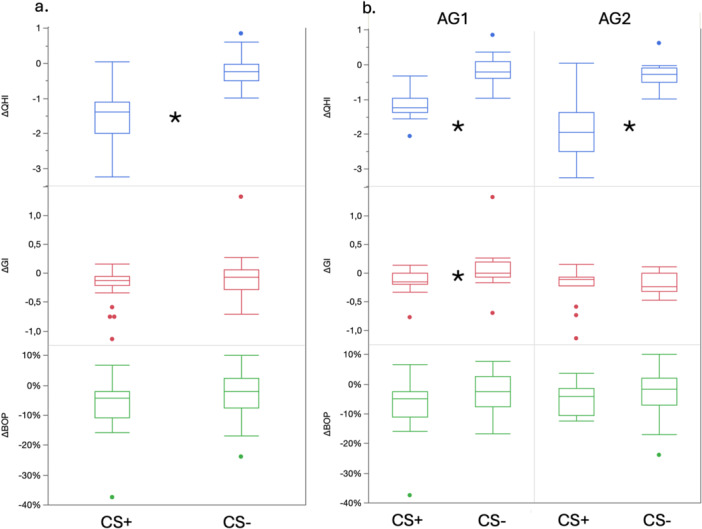
Box plots showing the differences between the aggregated baseline values and the two weeks values for the cleaning splint (CS+) and the cleaning without cleaning splint (CS‐). (a) overall cohort, (b) Age group 1 (AG1) and age group 2 (AG2). Each difference was calculated as 2W‐BL. *p < 0.05 by Mann–Whitney *U* test.

Pairwise comparison of baseline (T0 + T2) and 2 W values (T1 + T3) showed a significantly higher QHI reduction (∆QHI) with CS than without. The same effect was observed for AG1 and AG2. However, there was no statistically significant difference in the reduction of GI and BOP, except for a greater GI reduction with the test intervention in AG1 (Figure 3). test intervention in AG1 (Figure 3).

Further investigation using a random effect model demonstrated a significant QHI reduction with CS compared to conventional brushing (Table 3a), while apraxia scores had no influence and showed no interaction with CS use (*p* = 0.572). Higher baseline QHI was associated with greater QHI reduction (Table 3a).

**Table 3:** Regression analysis of potential effects of the cleaning splint in combination with different variables, co‐variables and interactions on (a) ∆QHI, (b) ∆GI, and (c) ∆BOP as dependent variables.

	Dependent variable	Term	Estimate	L‐CI	U‐CI	*p* value
a.	∆QHI	Intercept	0.981	−5.115	7.076	0.744
		Cleaning splint	−0.620	−0.731	−0.510	**< 0.001**
		QHI baseline	−0.434	−0.628	−0.239	**< 0.001**
		Apraxia score	−0.009	−0.117	0.098	0.854
		Apraxia score × cleaning splint	0.052	−0.022	0.126	0.164
b.	∆GI	Intercept	0.385	−0.001	0.771	0.051
		Cleaning splint	−0.084	−0.156	−0.012	**0.023**
		GI baseline	−0.442	−0.651	−0.234	**< 0.001**
		MEDAS	−0.052	−0.09	−0.004	**0.033**
		PSS	−0.002	−0.020	0.015	0.733
		PSS × cleaning splint	0.004	−0.012	0.020	0.629
		MEDAS × cleaning splint	0.022	−0.024	0.068	0.346
		PSS × MEDAS	0.003	−0.006	0.012	0.559
c.	∆BOP	Intercept	0.088	−0.021	0.197	0.112
		Cleaning splint (CS)	−0.019	−0.028	−0.009	**< 0.001**
		BOP baseline	−0.620	−0.757	−0.483	**< 0.001**
		MEDAS	−0.00	−0.022	0.006	0.221
		PSS	0.000	−0.004	0.005	0.960
		PSS × cleaning splint	−0.003	−0.005	−0.001	**0.001**
		MEDAS × cleaning splint	0.001	−0.004	0.007	0.635
		PSS × MEDAS	−8.4 × 10^−5^	−0.002	0.003	0.949

*Note:* Significant *p*‐values are presented in bold.

Abbreviations: BOP, bleeding on probing; GI, Gingiva Index; L‐CI, lower confidence interval; M‐CI, upper confidence interval; MEDAS, Mediterranean Diet Adherence Screener; PSS, perceived stress score; QHI, Quigley–Hein Index.

Similarly, GI reduction was significantly influenced by CS when baseline inflammation was introduced as an independent variable. Diet quality (MEDAS) was significantly associated with reduced gingival inflammation, while PSS showed no significant association with gingival inflammation (Table 3b).

Similarly, BOP reduction was slightly affected by the CS, mainly influenced by baseline BOP. Notably, while PSS alone had no effect, its interaction with CS use was associated with reduced BOP, though less effectively than CS use without stress.

### 3.5 Evaluation of the Cleaning Splint in Context of Missing Adjacent Teeth and Tooth Region (Front vs. Side)

At interdental space level, CS use was associated with a significantly reduced plaque accumulation. Plaque reduction was lower in spaces with interdental gaps, but this was mitigated by a significant interaction between CS use and gap presence, indicating enhanced CS effectiveness in such areas (Table 4a).

**Table 4:** Regression analysis of potential effects of the cleaning splint in the context of (a) presence of adjacent teeth and (b) the tooth region on ∆QHI as dependent variable.

	Term	Estimate	L‐CI	U‐CI	*p* value
a.	Intercept	2.599	−10.525	15.724	0.688
	Cleaning splint	−0.767	−0.971	−0.563	**< 0.001**
	Gap present	0.107	0.039	0.175	**0.002**
	Gap present × cleaning splint	−0.126	−0.188	−0.065	< **0.001**
	QHI_baseline	−0.627	−0.684	−0.57	**< 0.001**
	Apraxia score	−0.027	−0.267	0.213	0.817
	Apraxia score × cleaning splint	−0.005	−0.245	0.235	0.964
b.	Intercept	5.126	−8.016	18.269	0.430
	Cleaning splint	−0.718	−0.922	−0.515	**< 0.001**
	Front teeth	−0.070	−0.122	−0.018	**0.008**
	Front teeth × cleaning splint	−0.0191	−0.070	0.032	0.462
	QHI_baseline	−0.608	−0.663	−0.554	**< 0.001**
	Apraxia score	−0.075	−0.316	0.164	0.523
	Apraxia score × cleaning splint	0.0497	−0.190	0.289	0.674

*Note:* Significant *p*‐values are presented in bold.

Abbreviations: L‐CI, lower confidence interval; M‐CI, upper confidence interval.

Regarding tooth area, the CS remained effective overall. Front teeth showed slightly greater plaque reduction regardless of treatment, but no significant interaction between CS use and tooth area was found, suggesting the splint neutralized potential effects of the tooth area on plaque reduction (Table 4b).

## 4 Discussion

Cleaning splints offer a promising approach to enhancing interdental brush efficacy for biofilm removal. The null hypothesis of this study was that the use of a customized cleaning splint in combination with interdental brushes would not result in greater plaque reduction or improvements in periodontal parameters compared to the use of interdental brushes alone. The present findings indicate that the adjunctive use of a cleaning splint resulted in a significantly greater reduction in plaque accumulation (QHI) compared with conventional interdental brush use alone, suggesting that the null hypothesis can be rejected with regard to plaque reduction.

In addition to plaque reduction, significant reductions were observed in gingival inflammation (GI) and bleeding on probing (BOP) over the study period. However, the effects on inflammatory parameters were less consistent, and further studies with larger sample sizes are needed to confirm these observations. Subgroup analyses by age indicated the most pronounced for QHI, with a significantly greater reduction than conventional interdental cleaning, consistent across both age groups. While GI and BOP also decreased, statistical significance was observed only in the younger age group. However, these analyses should be interpreted with caution due to the limited sample size, short study period and exploratory nature of these comparisons.

Further analysis confirmed that the cleaning splint was effective regardless of motor coordination impairments, as apraxia scores showed no significant influence on plaque reduction. Interestingly, within the regression model, also diet quality—estimated by MEDAS—was significantly associated with reduced gingival inflammation. MEDAS is a validated short diet screener, estimating the similarities of patient diet to the Mediterranean diet and therewith to a healthy plant‐based diet pattern. It has been used in a number of studies in different medical fields (Li et al. 2020; Mieziene et al. 2020; Young et al. 2021). An inverse correlation to inflammatory parameters within studies that investigated the effect of the Mediterranean diet on gingivitis has been demonstrated (Bartha, Exner, Meyer, et al. 2022; Bartha, Exner, Schweikert, et al. 2022). Research suggests that dietary habits can influence periodontal parameters, indicating a potential benefit of a plant‐based whole‐food diet in promoting oral health (Baumgartner et al. 2009; Woelber et al. 2016, 2019).

Perceived stress (PSS) had no direct effect on gingival inflammation. However, an interaction between PSS and the cleaning splint was observed for BOP, suggesting that stress may modulate plaque control effectiveness when the splint was used. A positive relationship may exist between stress, mental health disorders, and periodontal disease, which can lead to behavior modifications such as poor oral hygiene practices. Additionally, stress has immunomodulatory effects, influencing immune cell numbers and function, as well as promoting proinflammatory cytokine production (Ball and Darby 2022).

At the interdental space level, the presence of gaps was associated with reduced plaque reduction, with the cleaning splint significantly mitigating this effect. Additionally, while plaque reduction was slightly higher in the anterior region, the cleaning splint remained effective across all tooth areas.

The findings of this clinical study align with those of Passia and Kern, who demonstrated that cleaning splints improved access to proximal surfaces in double crown prostheses and implants (Kern 2012; Passia and Kern 2021). Compared to their prosthesis‐specific design, the cleaning splint in this study is adaptable to all dental conditions and features triangular, funnel‐shaped openings for easier interdental access. The funnel‐shaped opening of the cleaning splint may aid interdental brush insertion by guiding the brush into the approximal space. Its design allows access to mesial and distal surfaces while potentially reducing gingival trauma.

The efficacy of the cleaning splint was evaluated in a preliminary in vitro study using typodont models. Results demonstrated that the use of a cleaning splint did not hinder the effectiveness of interdental brushes (IDBs) and significantly enhanced mean approximal cleaning efficacy at tooth surfaces adjacent to edentulous spaces (*p* = 0.001). Notably, cleaning efficacy was most pronounced at the gingival level (Rues et al. 2024). The study has several limitations that shall be addressed. (1) Each intervention period lasted only 2 weeks, which does not allow conclusions about long‐term adherence. Also, participants had only a short familiarization period with the cleaning splint, and handling efficiency may improve with longer use. Moreover, effects on inflammatory parameters might also be more reliably detectable in longer observation periods. (2) The study was designed as an exploratory pilot trial, and the sample size was determined by feasibility rather than an a priori power calculation. Hence, results should be interpreted as hypothesis‐generating. (3) Due to the crossover design and clinical workflow of the study, the examiner was not blinded, which may have introduced observer bias. In addition, participants were aware of the intervention, potentially introducing performance bias. (4) The study was conducted in a university‐based population of patients with periodontitis, which may limit the generalizability of results to other patient groups. (5) No microbiological analyses were performed, so potential changes in the composition or pathogenicity of the interdental biofilm remain unknown. (6) Toothbrush type was not standardized, as the primary focus of the study was on interdental plaque removal. Although participants were instructed to maintain their habitual brushing routines, some variability in overall plaque levels due to brushing differences cannot be excluded.

An additional limitation to the above mentioned of the present study relates to the practical aspects of implementing customized cleaning splints in daily clinical practice. The fabrication of an individual splint is associated with additional costs (approximately 360 € per device), which may limit widespread use and patient acceptance. Furthermore, long‐term durability was not systematically evaluated in this study. Based on clinical experience, the splint can be used until material fatigue or damage occurs; however, no predefined replacement interval was established. Since the splint is only worn for short periods during oral hygiene procedures and is therefore subjected to less mechanical stress than occlusal splints for bruxism, its service life may potentially be longer. This requires further evaluation in long‐term studies.

Patient compliance may also be influenced by maintenance requirements. The splint should be cleaned after each use with clear water and, if necessary, mild detergent, and stored in a dedicated splint box to ensure hygienic conditions. These additional handling steps may affect adherence and should be considered when interpreting the clinical applicability of this approach. Future studies should further investigate long‐term cost‐effectiveness, durability, and patient‐reported outcomes related to handling and maintenance.

On the other hand, a strength of this study is its randomized crossover design, which allowed each participant to serve as their own control and minimized inter‐individual variability. The clinical setting, combined with standardized oral hygiene instructions and controlled washout periods, ensured a high degree of protocol adherence. The predefined age stratification (≤ 40 and ≥ 65 years) was selected to explore potential age‐related differences relevant to interdental cleaning. Older individuals are more likely to exhibit reduced fine motor skills and increased tooth loss, both of which were examined in the analyses (Apraxia test and gap‐specific evaluation). The use of both objective clinical indices (QHI, GI, BOP) and validated instruments to assess potential modifying factors on inflammation or cleaning quality, such as diet quality, stress levels, and motor skills, provided a comprehensive evaluation of the intervention's effects.

As the demand for enhanced oral hygiene care grows, cleaning splints could also be a valuable tool for patients in nursing homes and other vulnerable groups, especially when supported by personalized instruction and assistance from caregivers or nurses (Zenthöfer et al. 2013, 2014). However, further studies are necessary to assess their effectiveness and practical implementation.

## 5 Conclusion

This randomized clinical crossover pilot study indicates that customized 3D‐printed cleaning splints may enhance the efficacy of interdental brushes, as their adjunctive use resulted in a significantly greater reduction in plaque accumulation in the present study and showed beneficial effects across different age groups. Gingival inflammation and bleeding on probing were also reduced, particularly in younger participants. The findings further suggest potential benefits in anatomically challenging interdental situations, including areas with missing adjacent teeth. These findings support the potential of cleaning splints as a practical adjunct for improving interdental hygiene. Long‐term clinical trials are warranted to confirm their impact on periodontal health and evaluate patient adherence in daily use.

## Author Contributions

Valentin Bartha, Marcia Spindler, Stefan Rues, and Andreas Zenthöfer were responsible for the conceptualization and methodology of the study. Marcia Spindler and Valentin Bartha supervised the concept of the study. Marcia Spindler and Valentin Bartha wrote the manuscript. Theresa Becker conducted the clinical study. Patient recruitment was carried out by Theresa Becker, Valentin Bartha, and Marcia Spindler Data entry was performed by Theresa Becker. Data analysis and statistical evaluation were performed by Valentin Bartha, Kevin Richter, and Stefan Rues. Resources were provided by Stefan Rues, Diana Wolff, and Peter Rammelsberg. All authors reviewed, revised, and approved the final version of the manuscript.

## Funding

The authors have nothing to report.

## Ethics Statement

This study was approved by the Ethics Committee of the Faculty of Medicine, Heidelberg University (Approval No. S‐387/2023).

## Conflicts of Interest

Andreas Zenthöfer and Stefan Rues own a European patent (non‐commercial) on the investigated cleaning splints. The other authors declare no conflicts of interest.

## Data Availability

The datasets generated and analyzed during the current study are available from the corresponding author upon reasonable request.
